# Supplementary material for: Are carbon emissions trading and green financial instruments synergistic? -Comprehensive quantitative research based on content analysis
Source: PLoS One. 2024 Mar 7;19(3):e0298601. doi: 10.1371/journal.pone.0298601 (PMC10919601; doi:10.1371/journal.pone.0298601)
Supplement: S1 Data — (ZIP) [file pone.0298601.s001.zip › Data/地级市Z_F工作报告.zip!/╕ú╜¿/╧├├┼╩╨/└┤╫╘╣1⁄2╓┌║┼í╛┬φ┐╦╩2╛▌═°í┐_2016.docx]

各位代表:

　　现在，我代表市人民政府向大会报告工作，请予审议，并请市政协委员和列席人员提出意见。

　　一、2015年和“十二五”工作回顾

　　2015年，我们在市委的正确领导下，深入贯彻党的十八大和十八届三中、四中、五中全会精神，认真落实习近平总书记系列重要讲话和对福建、厦门工作的重要指示精神，全面实施美丽厦门战略规划，深化改革开放，突出创新驱动，加快产业转型、城市转型、社会转型，较好完成全年各项任务。多数经济指标增幅逐月回升，完成地区生产总值3466亿元，增长7.2%。全社会固定资产投资1896.5亿元，增长20.6%；财政总收入1001.7亿元,增长10.2%，其中，地方级财政收入606.1亿元，增长11.5%，这些指标增幅均位居全省前列。城乡居民人均可支配收入增幅均高于同期经济增速；居民消费价格上涨1.7%；万元生产总值耗电、耗水分别下降4.5%和3.6%，年度节能减排任务全面完成。

　　一年来主要工作和成效:

　　（一）加快转变发展方式，经济发展质量效益不断提高

　　产业转型步伐加快。出台促进工业稳增长11条、加快智能制造发展10条、大众创业万众创新35条、扶持小微企业8条等措施。制造业质量竞争力指数88.99，工业化信息化融合发展指数90.49，居全国前列。联芯、天马微电子二期、三安集成电路、电气硝子等一批龙头项目进展顺利，建成全国最大LED外延芯片制造基地。实施中国制造2025行动计划，启动24个机器换工项目，技改投资占工业投资近70%。软件信息业务收入增长23%，生物与新医药产业链产值增长17.8%，海洋产业增加值增长10.8%，文化产业增长20%，网络零售额增长60%。接待境内外游客6035.8万人次，增长13%；旅游总收入832.3亿元，增长15.2%。国际投资论坛等四项展会获亚洲杰出展会奖。海港货物和集装箱吞吐量分别增长2.5%和7.1%，增幅居沿海主要港口前列。金融机构本外币存、贷款余额分别为8876亿元和7567亿元，增长16%和13.9%。在国家现代服务业综合试点城市复评中名列第一。高标准农田、现代设施农业、菜篮子基地建设加快，41家农业产业化龙头企业实现产值362亿元，销售收入349亿元。

　　产业平台建设加快。实施主体功能区战略，启动建设46平方公里的同安、翔安两个高新技术产业基地和8平方公里的丙洲、美峰两个现代服务业基地，规划建设环东海域东部新城、翔安南部新城、马銮湾新城，强化产业项目准入遴选。软件园三期、清华紫光集成电路产业园、生物医药港等产业功能区进展顺利，岛内老工业厂房改造提升加快。软件园二期创新社区建设初见成效，实现产值483亿元，增长20.1%。火炬高新区“一区多园”建设加快，规上工业产值占全市41.4%。省内协作经济区有序推进。

　　大众创业万众创新蓬勃发展。成为小微企业创业创新基地城市示范，获中央财政9亿元专项支持，构建29个双创集聚片区和92个众创空间。获国家科技计划立项420个。新建5个市级公共技术服务平台，新认定重点实验室、工程中心10家。实行“研究院+企业+地方”三方共建，设立军民融合协同创新研究院等产学研平台。高新技术企业突破1000家，规上高新技术产业产值3315.9亿元，占规上工业产值65.9%。全市各类专利授权1.1万件，每万人拥有有效发明专利14.13件。落实“互联网+”行动计划，获批国家信息消费示范城市。加快各类人才培育引进，2300多人入选“海纳百川”人才计划。

　　（二）加快打造国际一流营商环境，改革开放引向深入

　　自贸试验区建设取得阶段性成果。全面落实李克强总理考察厦门时所作“先行先试、敢闯敢试，显现特色、活力四射”重要指示，实施52项制度创新，其中全国首创18项。“一照一码”在全国复制推广，率先建设国际贸易单一窗口，创新“三互”口岸监管机制，启用关检一站式查验平台。实施外资“准入前国民待遇+负面清单+备案管理”模式，引进企业7584家，注册资本1102亿元。扩大服务业开放，加快金融改革创新，跨境电商、融资租赁、航空维修、文化保税等新兴业态快速发展。成立国际商事仲裁院、国际商事调解中心、两岸知识产权智库，承接253项省级行政许可事项，法治化服务环境加速形成。

　　重点领域改革实现新突破。深化行政审批制度改革，公布实施权力清单、责任清单和公共服务清单，市级权力事项精简51.3%。“多规合一”改革成为全国可复制推广样板。成立不动产登记局。基本完成事业单位分类。进一步完善商事登记制度，强化事中事后监管，健全征信体系，商事主体年报公示率93.3%。推行一系列国资国企改革，19家市属国企资产增长13.8%，利润增长5.3%。设立产业引导基金和城市发展基金。获批国家专项建设基金86.5亿元，占全省31%。发行90亿元地方政府债券，启动首批10个PPP试点项目。300多个项目纳入政府购买服务范围。获批内贸流通体制改革试点，城市配送网点覆盖率超60%。

　　国际化水平持续提升。合同利用外资41.6亿美元，增长45.9%；实际利用外资20.9亿美元，增长6.2%。走出去步伐加快，境外投资成倍增长，突破20亿美元。进出口总额832.9亿美元，增幅高于全国平均水平。开通厦蓉欧、中亚国际货运班列，时间比海运缩短一半，开辟了台湾及东南亚地区货物经厦门往返欧洲中亚的便捷通道。厦航开通厦门直飞阿姆斯特丹、悉尼洲际航线。实行外国人72小时过境免签，空港口岸实行全天候通关。获准实施离境退税政策。中国-东盟海洋合作中心落户厦门。邮轮母港出入境艘次和人次均增长2倍。成功举办厦洽会、首届中国-新西兰市长论坛、国际海洋周、国际时尚周、国际纪录片大会、中国-东盟邮轮经济城市合作论坛、第八届世界福建同乡恳亲大会、第十届世界同安联谊大会。

　　对台交流融合不断深化。海沧青创、一品威客、宸鸿科技被授予海峡两岸青年创业基地。设立台企快车服务中心、两岸企业搭桥中心，为台商投资提供一站式服务。厦台海运快件常态化运作。厦门口岸台湾食品进口批次占大陆三分之一。新设台资项目（含转第三地）增长1.2倍，实际利用台资增长61.2%。率先开展对台跨境人民币贷款，企业提款金额占大陆试点总量85%。跨海峡人民币代理清算金额累计537.1亿元。率先试行卡式台胞证。厦金“小三通”运送旅客164万人次，增长16.2%。成功举办第七届海峡论坛，工博会、文博会、两岸乐活节、民间艺术节等活动影响力增强。

　　（三）加快推进城乡建设，承载力和宜居度持续提升

　　城市建设有序推进。加快重大基础设施互联互通，地铁、翔安机场、海沧新港区、城市快速路系统加快建设。建筑业总产值1106.9亿元，增长25.5%。成为全国唯一海绵城市、地下综合管廊双试点城市，获中央财政20多亿元专项支持。已建成纳入综合管廊的市政管线210公里。规划建设35个公共停车场。建成公共自行车道205公里、站点435个。长泰枋洋水库引水隧道全线贯通，莲花水库主坝顺利完工。基本完成岛内外自来水厂清水互补环状供水体系。完成37处城市易涝点改造。生活垃圾分类处理厂、西部垃圾焚烧发电厂一期等投入使用。目前国际上输送容量最大的100万千瓦柔性直流变项目投入运营。

　　城市管理更加精细化。推进城市综合管理试点，完善源头管控，开展占道经营、违章停车、油烟噪音扰民等12个专项整治，拆除违建913.6万平方米。新增优化公交线路75条，增投出租车600部。53家运输企业、879辆渣土车纳入管控平台实时监控。成为餐厨废弃物资源化利用和无害化处理试点城市，生活垃圾无害化处理率100%。连续三届入选中国十大智慧城市，统一实名认证服务和预约挂号两个平台获世界互联网大会表彰。鼓浪屿整治提升取得成效，53个申遗核心要素加快修缮，历史文化魅力日益彰显。

　　美丽乡村建设持续深入。统筹推进新型城镇化，落实支农强农惠农政策，转移农村富余劳动力1.2万人，农村居民可支配收入居全省首位。开展造血式扶贫，老区扶建资金由200万元提高到1000万元。实施新村建设、旧村改造、老区山区村改造项目15个，建设移民美丽家园11个。农村有线电视入户率99.9%。探索生态资源保护、产业形态丰富、百姓增收致富良性循环机制，院前社闽台生态文化村建设取得成效，洪塘村、军营村获评中国最美休闲乡村，14个村庄获评省级美丽乡村建设示范典型村。

　　城乡生态环境持续优化。通过国家生态市考核验收，14个涉农镇街均获评国家级生态镇，93.6%行政村获评省级生态村。专项整治工业污染，淘汰高排放产业，加大黄标车淘汰力度，加强城市道路、建筑施工、堆场料场扬尘治理，空气质量优良率99.18%，在全国74个重点城市中排名第二。落实水污染防治行动计划，饮用水源水质达标率100%。推广许溪、过芸溪整治经验，全面开展九条小流域综合治理。完成环筼筜湖30个和杏林湾52个排污口截污、环岛路42处排洪沟截流，城镇污水集中处理率93.4%。编制海洋生态红线，完成海域清淤1200万立方米。造林绿化950公顷，新增城市园林绿地414公顷，完成水土保持治理667公顷。

　　（四）加快完善基本公共服务，民生保障更加有力

　　社会事业繁荣发展。新改扩建中小学项目25个，开建公办幼儿园19所，分别新增学位2万个和5000个。获批全国三个校园足球改革试验区之一。21所中职学校与185家企业建立校企合作实训平台。第二医院三期投入使用，复旦中山厦门医院、厦大附属翔安医院等启动建设，弘爱医院等社会资本办医项目进展顺利。流动人口计生基本公共服务均等化和社会融合示范试点取得成效。国家海峡版权交易中心挂牌成立。国内规划面积最大的儿童公园一期、二期建成开园。成为全国体育产业联系点城市。厦门国际马拉松连续九年获评国际田联路跑金牌赛事，促进了群众性体育运动蓬勃开展。山海协作、对口帮扶工作扎实推进，老龄、残疾人工作不断加强，妇女儿童合法权益得到保障，民族团结宗教和睦，人防海防、档案方志工作全面进步。

　　民生改善力度加大。总投资2.4亿元的14件为民办实事项目全部完成。全年新增就业18万人。实施新一轮职业技能培训补贴政策和高校毕业生创业引领计划。企业最低工资标准调高至每人每月1500元。设立规范化镇街劳动仲裁派出庭和社区劳资纠纷综合调解室。城乡居民基本医疗保险筹资财政补助标准提高至470元，高出国家标准90元。调整医疗服务价格1108项，全面取消耗材加成，社区诊疗报销比例提高至93%以上。在全国率先实现慢性病患者分诊20%目标。降低门诊和住院医疗费社会统筹医疗基金起付标准，每年可减轻参保人员负担1.5亿元。在北上广三地24家医院试点医保就地一站式结算。企业退休人员基本养老金提高至月人均3118元，居全国前列。建成366个居家养老服务站。完成老旧小区改造19个，开工建设保障性安居工程5107套，基本建成7290套。社会福利和慈善事业持续发展，气象、防震、防灾减灾工作稳步提升。

　　共同缔造成效显著。全国社区治理创新工作会议在厦举行，民政部等在全国推广共同缔造经验。建成镇街、村居全覆盖网格化信息平台，社区普遍成立社企共建理事会。完成村居换届选举。加快“三社联动”，打造143个典范村（社区），持证社会工作者占全省42%，登记备案社会组织3675个。强化危化品、燃气、电梯等重点领域安全监管，安全生产各项指标大幅下降，形势总体平稳向好。深化建设平安厦门，在全国率先实施多元化纠纷解决机制地方性法规。建成“阳光信访”信息平台，来信来访批次、人数分别下降34.8%和23.5%。开展打黑除恶、围点整治等专项行动，八类严重暴力刑事立案数下降20%。顺利通过全国双拥模范城创建工作考评。荣获全国文明城市“四连冠”。

　　政府自身建设不断加强。深入开展“三严三实”专题教育，严格落实中央八项规定精神和国务院约法三章，强化效能建设、行政监察和审计监督。基本完成各级行政服务中心标准化建设，办事群众满意率99%。清理规范涉企收费，企业减负超120亿元。分类管理退税企业，办理退税412.2亿元，增长17.2%；网上办税占92%。全市“三公”经费支出减少16.9%。全面完成“六五”普法。坚持依法行政，法治政府评估保持全国前列。提请市人大常委会审议法规草案6件，制定规章2件；修改规章17件、废止13件。在全国率先建立规范性文件网上审查系统，全面落实行政自由裁量权基准制度。主动公开政府信息3.5万件。政府与法院、检察院、工会互动机制不断完善。自觉接受人大法律监督、工作监督和政协民主监督，认真办理市人大代表建议337件、市政协提案497件，办复率均为100%，满意率分别为97.9%和95.8%。

　　各位代表！“十二五”期间，我们牢牢把握中央支持福建加快发展的重大历史机遇，扎实推进产业升级、体制创新等十大行动计划，经济社会发展取得明显进步。一是综合实力得到新提升。地区生产总值年均增长10.6%，人均GDP 1.4万美元。规上工业总产值突破5000亿元，年均增长12.7%。财政总收入突破1000亿元，年均增长15%，地方级财政收入年均增长16%。全社会固定资产投资累计7277.7亿元。三次产业结构从1.0∶51.1∶47.9调整为0.7∶43.8∶55.5。平板显示、金融服务、旅游会展3条产业链产值分别突破千亿。二是城乡建设迈出新步伐。厦漳跨海大桥、龙厦铁路、厦深铁路、厦成高速建成通车，全国首个自动化码头正式运营，完成厦漳港口一体化整合。城市建成区绿化覆盖率41%，人均公园绿地11平方米，成为国家森林城市。三是改革开放获得新突破。两岸交流合作综合配套改革先行先试92项政策，“一区三中心”建设初显成效。完成新一轮政府机构改革，行政审批事项精简64.6%，名列中国服务型政府十佳城市前茅。实际利用外资92.4亿美元，27家世界500强企业在厦投资或增资扩产。外贸综合竞争力居全国百强城市第五位，进出口额占全省49%。四是改善民生取得新成效。全市财政一般预算70%以上投入民生和社会事业。新增城镇就业75.2万人，转移农村劳动力8.7万人。实现城乡义务教育完全免费和中等职业教育免学费。率先实现大病医保城乡全覆盖，统一城乡居民基本医疗保险政策和最低生活保障标准。开展养老服务业综合改革试点。建成保障性安居工程5.5万套。五是社会治理再上新台阶。全面构建“横向到边、纵向到底、协商共治”社区治理体系，实施“以奖代补”项目932个，覆盖全市269个社区。成为全国社区治理和服务创新实验区，获评全国和谐社区建设示范城市。连续三届获评全国社会治安综合治理优秀城市。荣获全国双拥模范城“八连冠”。

　　各位代表！成绩来之不易，这是在市委坚强领导下，在人大代表、政协委员大力支持和有效监督下，全市人民齐心协力、艰苦奋斗的结果。我代表市人民政府，向全市人民，向全体人大代表和政协委员、各民主党派、工商联、各人民团体、无党派人士、离退休老同志和社会各界人士，向中央和省驻厦单位、驻厦部队、武警官兵、公安干警，向来厦投资者和劳动者，向关心支持厦门发展的港澳同胞、台湾同胞、海外侨胞和国际友人，表示衷心感谢！

　　我们也清醒地看到存在的困难和问题，主要有:地区生产总值、固定资产投资、社会消费品零售总额、外贸进出口总额等4项指标未能达到“十二五”预期目标，经济下行压力依然较大；龙头企业不多、带动力不足，发展后劲和创新能力亟待增强；违章停车、流动摊贩等现象仍然存在，部分路段在一些时点存在拥堵，城市综合管理还需加强；基本公共服务均等化水平有待提高，教育、医疗、养老等需进一步改善，群众的一些迫切需求尚未得到有效解决，公共安全仍有隐患；政府职能转变还不到位，有的部门回避矛盾、推诿扯皮，有些人员消极作为、不敢担当，个别人员甚至违纪违法。我们要正视这些问题，采取有力措施，认真加以解决。

　　二、“十三五”时期展望

　　按照市委十一届十一次全会的部署，我市“十三五”发展的指导思想是:高举中国特色社会主义伟大旗帜，深入贯彻党的十八大和十八届三中、四中、五中全会精神，以马列主义、毛泽东思想、邓小平理论、“三个代表”重要思想、科学发展观为指导，深入贯彻习近平总书记系列重要讲话及对福建、厦门工作的重要指示精神，坚持“四个全面”战略布局，坚持发展第一要务，全面落实中央支持福建、厦门进一步加快经济社会发展的重大决策部署，统筹推进经济、政治、社会、文化、生态文明建设和党的建设，以加快产业、城市和社会转型为主线，以提高经济发展的质量和效益为中心，着力创新发展、协调发展、绿色发展、开放发展、共享发展，形成适应经济发展新常态的体制机制和发展方式，率先全面建成小康社会，争当“五大发展”示范市，建成美丽中国典范城市，为建设机制活、产业优、百姓富、生态美的新福建作出新的更大贡献。

　　《厦门市国民经济和社会发展第十三个五年规划纲要（草案）》明确经济发展、发展动力、民生福祉、生态文明四大类30项主要目标，提出比全国提前三年实现地区生产总值和城乡居民人均收入比2010年翻一番；到2020年，人均地区生产总值和人类发展指数(HDI)达到中等发达国家水平。

　　今后五年，经济社会发展主要战略任务是:

　　（一）以创新驱动加快产业转型。围绕提升产业竞争力和带动力，构建“5+3+10”现代产业体系，培育形成半导体和集成电路、软件信息服务等一批千亿产业链（群）。大力实施创新驱动发展战略，建立以企业为主体、市场为导向、产学研用相结合的技术创新体系。大力实施质量强市战略，构建先进制造业、现代服务业、现代农业、节能减排、社会管理和公共服务等领域的厦门标准体系。整合提升现有产业园区，新建成两大高新技术产业基地和两大现代服务业基地。以国有企业、财税体制、投融资体制等为重点，整合要素资源，扶持优势产业。规上工业增加值年均增长10%左右，第三产业增加值占GDP力争突破60%。全社会研发投入占GDP达4%，科技进步贡献率达65%，高新技术产业产值占规上工业产值达70%。

　　（二）以统筹协调加快城市转型。大力实施大海湾、大山海城市发展战略，通过“多规合一”和主体功能区规划，完善一岛一带多中心和山海城相融合的城市空间格局，统筹优化岛内中心城区和岛外新城建设，加快岛内外一体化、厦漳泉同城化，实现一体发展、优势互补、合作共赢。通过完善基础设施，提升城市系统性、协调性和承载力、宜居度。统筹建设大路网、大港口、大物流，建成福厦高铁、厦深高铁和4条地铁，形成城市快速路网；港口货物吞吐量达3.4亿吨，集装箱吞吐量达1300万标箱；空港旅客年吞吐量2900万人次，翔安机场投入使用；建立大平台、大数据，初步建成国际知名智慧城市。

　　（三）以绿色低碳推进可持续发展。大力实施大花园城市发展战略，严守生态控制线，涵养保育山体，综合治理流域，修复近海生态，巩固提升水环境和空气质量。节约集约利用资源，发展循环经济。健全生态文明体制机制，加强环境监管能力建设。节能减排降碳和生态环境质量等主要指标保持全国领先水平，实现百姓富与生态美有机统一,走出一条生产稳健发展、生活富裕安康、生态优美宜人的文明发展成功之路。

　　（四）以开放合作提高国际化水平。主动融入全球经济体系，实施全方位对外开放战略，全面建设自贸试验区，建立与国际通行规则相衔接的体制机制，投资贸易显著便利化。加快推进“一带一路”战略支点城市建设，打造与沿线国家互联互通枢纽、经贸合作枢纽、海洋合作枢纽、人文合作枢纽。进一步拓展对外经贸，加快引进来与走出去，打造更具竞争力的总部基地。申办和引进一批国际知名、市场运作的高端会展和文体赛事，提升城市国际影响力。

　　（五）以包容共享加快社会转型。着力保障和改善民生，城乡居民收入稳步提高，基本公共服务均等化程度居东部发达城市前列，基本医疗保险参保人数达353万人，基本养老保险参保人数达254万人，每千名老人养老床位数40张。建设文化强市，文化产业成为支柱产业。共同缔造成为全社会自觉行动，市民文明素质和社会文明程度保持全国领先。人民合法权益得到切实保障，社会更加和谐稳定。

　　（六）以先行先试构建对台战略支点。深化两岸交流合作综合配套改革，服务两岸关系和平发展大局。创新两岸产业、贸易、金融等合作体制机制，拓展多层次多领域往来合作，厦金融合率先取得突破，成为台湾民众赴大陆就业定居首选。对台进出口贸易额突破100亿美元，对台跨境人民币业务量突破1000亿元，经厦门口岸赴台旅游人数、台湾经厦门口岸进出人数保持大陆首位。厦门成为两岸经贸合作最紧密区域、两岸文化交流最活跃平台、两岸直接往来最便捷通道、两岸同胞融合最温馨家园。

　　三、2016年主要工作

　　2016年，我们要牢牢把握当前和今后一个时期经济发展的大逻辑，牢固树立和贯彻落实“五大发展”理念，认识新常态、适应新常态、引领新常态，进一步解放思想、锐意改革、大胆创新，弘扬经济特区先行先试、敢闯敢试精神，推动实现更高质量、更有效率、更加公平、更可持续发展。

　　今年经济社会发展主要预期目标为:地区生产总值增长8.5%，力争9%；全社会固定资产投资增长18%，力争20%；财政总收入和地方级收入分别增长8%和6.5%；外贸出口增长3%；实际利用外资增长6%；社会消费品零售总额增长12%；城乡居民人均可支配收入分别增长8.5%和9%；居民消费价格涨幅控制在3%左右；城镇登记失业率控制在4%以内；完成国家和省下达的节能减排任务。

　　实现上述目标，必须扎实做好以下工作:

　　（一）推进供给侧结构性改革，着力增强发展新动能

　　面对深刻的供给侧、结构性、体制性矛盾，通过供给侧改革拓展空间，提高供给结构适应性灵活性，加快实现发展动力转换。

　　降低企业经营成本。落实好已出台20条降低企业成本措施，在降低社会保险费、财务成本、物流成本、制度性交易成本等方面，再出台一批新措施，使企业经营成本明显下降。支持企业提高市场竞争力，加大一业一策、一企一策精准帮扶，帮助有市场有前景的企业渡过难关、焕发生机。引导有竞争优势的龙头企业通过合资合作、产权流转、股权置换等方式并购重组。继续推进简政放权、放管结合、优化服务，大力推进并联审批、网上审批，进一步缩短时限、提高效率。深化商事登记制度改革，加强事中事后监管，营造公平诚信的市场环境。

　　切实扩大有效投资。更加注重选准投资方向、提高投资效益，下大力气补齐产业平台、工业技改、基础设施、生态环保、民生改善等领域短板，切实提高投资有效性和精准性。全面加快地铁1至4号线、翔安机场、新港区和港口集疏运体系建设，完善城市快速路系统。着力解决项目前期滞后和征地拆迁难题，加快一批在建项目进度，新开工一批前期较为成熟项目。进一步拓展融资渠道，扩大政府和社会资本合作力度，争取更多上级专项资金支持。

　　大力发展新技术、新业态、新模式、新产业。坚持高端引领，聚焦重点产业，主攻核心技术，加快推出一批新项目，培育一批新企业。鼓励和引导企业采用新技术、新工艺进行技术改造和产品升级。大力发展电子商务、教育培训、医疗保健、家政养老、体育健身、休闲娱乐等新业态。加快智慧城市和下一代互联网示范试点城市建设，发展物联网产业，培育发展个性化定制、众包设计、云制造等新模式。集中力量发展集成电路、LED、生物与新医药等战略性新兴产业，抓紧布局石墨烯、碳化硅等未来产业。

　　有序推进重点领域改革。加快内贸流通体制改革，推动流通信息化、标准化、集约化。深化国有企业改革，完善现代企业制度，加强国资监管。培育壮大民营经济，发展混合所有制经济。加大政府购买服务力度，提高公共服务供给质量和财政资金使用效率，推动“养人”向“养事”转变。加强资源配置，实施公共产品和服务价格改革，完善市场决定价格机制。全面实施不动产统一登记，推进农村土地承包经营权确权登记颁证。

　　（二）优化产业结构，着力提升产业竞争力

　　顺应经济从粗放向集约、从简单分工向复杂分工高级形态演进的趋势，从深层次推进结构调整，加快培育新的增长点。

　　抓好工业稳增长调结构增效益。培育生根型高新技术企业，加快联芯、三安LED二期等在建项目进度，确保电气硝子、天马微二期、开发晶等重大项目建成投产。引导协助宸鸿、友达等龙头企业配套完善产业集群。加快ABB工业中心、金龙汽车整合提升等项目建设。鼓励大博颖精等企业机器换工，争创国家智能制造试点示范基地。加快小微企业创业创新基地城市示范建设，推进思明龙山、湖里华美等双创集聚片区发展。完善金融保障体系，发挥产业基金放大效应，鼓励风投、创投等基金发展。优化创新创业环境，为企业提供早期办公、商业模式构建、团队融合、媒体资讯、教育培训、中介服务等，形成全方位服务双创生态体系。完善人才市场建设，引进、培养大批人才和研发团队。提升知识产权创造、运用、管理和保护能力。深入实施质量强市战略，推动品牌和标准化建设。

　　推动现代服务业优质高效发展。促进旅游与会展深度融合，打造国际旅游会展名城，创建国家旅游休闲示范城市。加强海洋研究，发展海洋相关产业。打造中转型港口，重点发展集装箱运输、国际航运服务、保税物流。培育金融租赁、消费金融等新兴业态，推动金融与城市建设、实体经济协同发展。大力发展软件信息、工业设计、服务外包、检验检测等生产性服务业。吸引优质企业来厦设立区域性、职能型总部。

　　做优做精现代都市农业。重点抓好高科技种苗业、设施农业、休闲农业、农产品精深加工业，促进一二三产联动发展。坚持最严格的耕地保护制度，推进高标准农田建设，加快土地整治。推进农业标准化，完善全过程监管体系，确保农产品从农田到餐桌的质量安全。大力培育专业大户、家庭农场、农民合作社等新型经营主体，支持龙头企业发展，提高农业经营集约化、规模化水平。认真做好农业普查工作。

　　（三）坚持扩大开放，着力提升对外开放水平

　　围绕打造国际一流营商环境，对标世界先进，接轨国际规则，以法治化、国际化、便利化营商环境促进开放型经济发展。

　　全面加快自贸试验区建设。以制度创新为核心，在全市复制推广自贸试验区改革经验。推动投资管理制度创新，完善负面清单管理，推出一批扩大开放新举措。深化贸易便利化改革，加快电子口岸建设，拓展单一窗口功能，完善大通关一体化平台。创新贸易监管制度，全面实施海关特殊监管区货物分类监管，推广内销货物分段担保监管。加快服务贸易开放，试点新型免税购物模式，做大跨境电子商务产业园，打造多样化进口商品展示销售中心。用好央行关于金融支持自贸试验区建设30条政策措施，扩大人民币跨境使用。打造企业注册、外贸管理、口岸通关、保税监管等公共信息平台，提高政府服务管理透明度。

　　努力提高招商引资水平。强化招商平台建设，深化项目前期策划，完善招商联动机制。组建高水平专业化招商队伍，大力开展上门招商、以商引商等。围绕产业发展方向，加大对知名外企、大型央企、重点民企和台湾百强企业的招商力度，引进填补产业链缺失项目和关键环节。鼓励支持有市场、有效益的企业增资扩产。实施走出去战略，积极参与国际竞争合作。

　　主动融入国家“一带一路”战略。推进与沿线国家和地区互联互通、经贸合作、人文交流。拓展海空航运服务功能，增辟航线、加密航班，整合海运快件，发展国际中转集拼业务。充分利用厦蓉欧、中亚班列，建设海陆联运大动脉，完善集疏运体系，使厦门成为“一带”“一路”联结点。加快建设跨境电商、人民币跨境结算和海外投资贸易服务平台。建好中国-东盟海洋合作中心，建成启用厦门领事馆区。把“9·8”投洽会办出新成效，保障好G20央行长财长副手会议，办好21世纪海上丝绸之路论坛、第五届南洋文化节。

　　（四）持续推进跨岛发展，着力提升城市功能品质

　　统筹生产、生活、生态三大布局，统筹岛内外一体化、差异化发展，增强城市生长性，促进城市布局优化、功能提升。

　　不断提升岛内。围绕增强承载力、提高宜居度，控制开发强度，降低建设密度，提升环境品质，完善城市功能。推动观音山、五缘湾等连片发展，完善鹭江道、火车站等周边配套，打造金融、商务、旅游会展、消费购物中心。加快老旧小区改造和城中村提升，推进湖边水库、高林等片区改造，建设一批停车场，完善公共配套。保护风景名胜、风貌建筑、历史文化街区，挖掘地域文化特质，强化鼓浪屿保护与管理，争创国家历史文化名城。

　　大力拓展岛外。全力加快建设同安、翔安两个高新技术产业基地和丙洲、美峰两个现代服务业基地，加快开发环东海域东部新城、翔安南部新城、马銮湾新城，做好规划，加强基础设施和学校、医院、文体等公共设施配套，抓紧引进一批带动力强的产业项目，聚集人气商气。加快海绵城市、地下综合管廊建设，推动海沧隧道、翔安机场快速路、前场铁路大型货场、北动车运用所等项目建设，开建莲花水库至西山水厂等3个水源连通工程。整治“四边三节点”，实施旧村改造、老区山区村建设和移民造福工程，改善农村生产生活环境。推进小城镇建设试点，加快实现农业转移人口市民化。

　　推动区域协作。加快厦漳泉同城化，推动设施互通、产业互补、资源共享。扎实推进厦门龙岩山海协作经济区、厦门泉州（安溪）经济合作区建设，推动临空临港产业园区规划和建设，完成长泰枋洋水库龙津溪枢纽工程和尚吉电站主体工程。深化与闽西南五市和闽粤赣十三市对接合作，做好对口支援新疆、西藏、宁夏、甘肃等地工作。

　　（五）扩大交流合作，着力促进两岸深度融合

　　发挥前沿平台优势，用好用足国家赋予的先行先试政策，推动两岸同胞往来更加便利、交流更加密切、利益更加交融。

　　增进经贸合作。加强与台湾集成电路、医疗养生、现代农业等产业对接合作。加快两岸新兴产业和现代服务业合作示范区、两岸区域性金融服务中心、东南国际航运中心和大陆对台贸易中心建设，组建两岸知识产权银行。加快海峡旅游服务中心建设，丰富对台双向旅游产品，持续打造海峡旅游品牌。对接高雄港、台中港，发展壮大海峡邮轮产业。率先启动厦台跨境电商监管互认试点。全面实施台湾输大陆商品快速验放。拓展海峡两岸青创基地内涵，大力引进台湾人才，鼓励台湾青年来厦创业生活。

　　增进交流交往。创新口岸通关监管，积极争取人员往来便利化等涉台审批权限。深化厦金多领域合作。加快五通客运码头三期建设，推动刘五店航道（金门段）投用。持续办好海峡论坛等涉台活动，扩大对台交流品牌影响力。加快海峡两岸教育交流与合作等基地建设。建立社区共建常态化机制，拓展两岸在文化艺术、宗亲联谊、民间社团等方面交流合作。

　　（六）坚持绿色发展，着力创建美好家园

　　提升国家生态文明建设示范市和先行示范区水平，进一步增强绿色发展竞争力。

　　加大节能减排力度。强化环境准入，高标准严控污染物排放总量。支持绿色清洁生产，推广节能产品和技术，鼓励企业技术装备更新改造。发展节能环保产业，建设充电设施，推广新能源汽车及装备。加强综合交通枢纽建设。实行公交优先，优化自行车专用道，鼓励绿色出行。提高建筑节能标准，推进公共建筑节能改造。

　　狠抓环境治理。实施清洁空气行动计划，重点整治汽车尾气、工业废气、扬尘污染、餐饮油烟。持续推进九条流域综合治理，强化畜禽养殖、农业面源、工业生产等污染防治。开展土壤污染治理和修复。推进海域清淤、岸线整治，开展湿地、沙滩生态修复，严控陆源污染物入海。治理水土流失，实施山水林田湖生态保护，筑牢生态安全屏障。完善城乡污水处理设施，加快环筼筜湖、五缘湾等片区排污口综合治理。推进东部、西部垃圾焚烧发电厂二期、后坑压缩中转站等垃圾处理基地建设，推行垃圾分类，倡导垃圾不落地。

　　健全生态文明制度。加快建设主体功能区，严格生态控制线管理，建立健全污染企业停产治理、淘汰退出机制，完善环境风险评估和生态补偿机制。扩大排污权有偿使用和交易，开展环境污染第三方治理。推行环境污染责任保险，加快企业环境信用评价信息平台建设。推广环保网格化监管，严肃生态环保责任追究，严厉打击环境违法行为。

　　（七）加快发展社会事业，着力推动公共服务公平均衡

　　从社会关注度高的问题入手，尽力而为、量力而行，增加供给、补齐短板，促进公平、普惠、可持续。

　　加快教育发展。推进重点教育基建项目建设，新增中小学学位2万个，完善随迁子女积分入学及小学、幼儿园招生政策。支持民办义务教育学校开展标准化建设。加强高中多样化办学改革试点。大力发展职业教育，优化专业设置，推进校企合作，扩大订单式人才培养。鼓励兴办国际学校。支持高校内涵发展，发挥高校人才培养、科技创新、文化传播和产业推动作用。营造尊师重教氛围，加强师德校风建设，完善教育质量评价体系。

　　提升医疗卫生服务。深化医药卫生体制改革，完善分级诊疗体系，加快公立医院改革，鼓励社会资本办医，不断满足多层次医疗需求。扩大“三师共管”慢性病分级诊疗服务范围，增加诊疗病种。加快已批医院建设，完善社区卫生服务站点，打造15分钟健康圈。推进省全科医生培训分中心建设，壮大全科医生和住院医师队伍。

　　完善医疗服务绩效第三方考评。加强医德医风建设，构建和谐医患关系。做好传染性疾病防控和应急救治。全面实施一对夫妇可生育两个孩子政策，促进人口长期均衡发展。

　　让市民共享文化成果。大力践行社会主义核心价值观，加强未成年人思想道德建设。弘扬嘉庚精神，汇聚侨心侨力。实施国有文艺院团及公益文化场馆改革试点。打造艺术精品，办好文博会、中国国际钢琴比赛等重大文化活动。加强文化产业公共服务平台建设，鼓励社会力量参与文化发展。深化文化惠民、文化下基层，推动文体设施向公众开放，广泛开展全民健身活动。

　　（八）提高民生保障水平，着力增强人民群众获得感

　　守住底线、突出重点、完善制度，更多改善低收入群众生活，更好维护社会大局稳定，让改革成果惠及全体市民。

　　积极支持就业创业。做好被征地农民和退养渔民、高校毕业生、城镇困难人员、转业退役军人等群体就业创业服务，推动充分就业。加快技师大师工作室项目建设，加强劳动技能培训，壮大高技能人才队伍。全面推广建筑领域预防劳资纠纷长效机制。推进工资集体协商，健全劳动监察和争议处理机制，深入构建和谐劳动关系。

　　完善社会保障体系。实行差别化医保支付政策，优化不同等级医疗机构报销比例，完善按病种、按服务单元、按项目等多种付费方式。修订补充工伤保险办法，完善大病补充医疗保险、临时救助和医疗救助等制度。全面实施机关事业单位养老保险制度改革，提高基本养老金。推广医养护结合，推进爱鹭老年养护中心、爱心护理院改扩建等项目，增设居家养老服务站，鼓励民办养老机构发展。开工建设保障性安居工程8082套，基本建成4538套。推行以公租房为主要方式的新型保障制度，健全准入和退出机制。强化社会保障兜底，城乡低保标准提高到每人每月610元，有效帮扶困难家庭。促进社会福利、慈善和妇女儿童、残疾人事业健康发展。

　　加强和创新社会治理。完善城乡社区网格化管理。推进“三社互动”，推广“社工+志愿者”服务模式。完善社区社会组织登记备案双轨制，建立区、街、社区三级社会组织孵化与服务体系。推进安全生产标准化建设，争创全国安全发展示范城市。建设救灾物资储备仓库，提升防灾减灾和应急处置能力。抓好食品安全城市创建试点，加强安全监管，打击假冒伪劣，维护市场秩序。推进交通改善工作，新增优化一批公交线路。持续创建平安厦门，积极构建“互联网+”群防群治新模式，健全公共安全体系。强化施工组织，严惩恶意阻工，保障重点项目。抓好社会矛盾源头治理，完善多元化纠纷解决机制，畅通群众诉求表达渠道。持续推进新一轮文明城市创建，打造全国公共文明行为典范城市。推动军民融合深度发展，探索完善政策拥军和国防动员机制，争创全国双拥模范城“九连冠”。

　　各位代表！完成今年目标任务、实现“十三五”良好开局，要求我们要切实加强政府自身建设。面对新常态，我们不能坐着等、站着看，必须加强学习、提高驾驭经济工作的能力；必须主动作为、积极为基层为企业提供优质服务，全面营造激发发展活力、再创特区辉煌的良好环境。

　　我们要持续提升政府公信力和执行力。增强担当意识、责任意识和大局意识，强化对重大决策、重要部署的执行力度，面对矛盾不退缩，直面问题不畏难，以过硬的作风抓落实、促发展，切实做到勤勉敬业、守土尽责。加强电子政务建设，构建综合高效的政务服务平台。坚持阳光用权，深化政务公开，以财政预决算、重大项目建设、社会公益事业等领域为重点，推进行政决策、执行、管理、服务和结果公开，保障广大群众的知情权、参与权、表达权和监督权。

　　我们要持续加强民主法治建设。进一步强化法治思维，将政府活动全面纳入法治轨道。坚持立改废释并举，制定实施地方性法规法定配套文件。健全综合执法机制，坚持严格规范公正文明执法。启动“七五”普法。加强新型智库建设，促进依法决策，认真履行重大行政决策公众参与、专家论证、风险评估、合法性审查、集体讨论决定等法定程序。自觉接受人大及其常委会法律监督、工作监督和政协民主监督，认真办理人大代表建议议案和政协提案。广泛听取各民主党派、工商联、无党派人士和各人民团体的意见建议。

　　我们要持续建设廉洁政府。巩固“三严三实”专题教育成果，严格落实中央八项规定精神和国务院约法三章，坚持不懈纠正“四风”。严格落实“一岗双责”，把纪律和规矩挺在前面，深入推进党风廉政建设和反腐败工作。坚持用制度管权管事管人，减少自由裁量权，做到政策公开、透明、可预期。严控“三公”经费，建设节约型机关。从土地出让、工程招投标、规划管理、财政资金使用等重点领域入手，全面加强财政监督、审计监督和社会公众监督，严厉查处违纪违法案件。

　　各位代表！新的一年，我们面临的任务艰巨繁重，肩负的责任重大光荣。让我们紧密团结在以习近平同志为总书记的党中央周围，在市委的坚强领导下，凝心聚力、扎实工作，更好发挥经济特区龙头带动作用，为加快建设美丽中国典范城市而努力奋斗！
